# Simulating Active Inference Processes by Message Passing

**DOI:** 10.3389/frobt.2019.00020

**Published:** 2019-03-28

**Authors:** Thijs W. van de Laar, Bert de Vries

**Affiliations:** ^1^Department of Electrical Engineering, Eindhoven University of Technology, Eindhoven, Netherlands; ^2^GN Hearing Benelux BV, Eindhoven, Netherlands

**Keywords:** active inference, free-energy principle, message passing, state-space models, Forney-style factor graphs

## Abstract

The free energy principle (FEP) offers a variational calculus-based description for how biological agents persevere through interactions with their environment. Active inference (AI) is a corollary of the FEP, which states that biological agents act to fulfill prior beliefs about preferred future observations (target priors). Purposeful behavior then results from variational free energy minimization with respect to a generative model of the environment with included target priors. However, manual derivations for free energy minimizing algorithms on custom dynamic models can become tedious and error-prone. While probabilistic programming (PP) techniques enable automatic derivation of inference algorithms on free-form models, full automation of AI requires specialized tools for inference on dynamic models, together with the description of an experimental protocol that governs the interaction between the agent and its simulated environment. The contributions of the present paper are two-fold. Firstly, we illustrate how AI can be automated with the use of ForneyLab, a recent PP toolbox that specializes in variational inference on flexibly definable dynamic models. More specifically, we describe AI agents in a dynamic environment as probabilistic state space models (SSM) and perform inference for perception and control in these agents by message passing on a factor graph representation of the SSM. Secondly, we propose a formal experimental protocol for simulated AI. We exemplify how this protocol leads to goal-directed behavior for flexibly definable AI agents in two classical RL examples, namely the Bayesian thermostat and the mountain car parking problems.

## 1. Introduction

The free energy principle (FEP) offers an ambitious theory for how biological agents perceive and interact with their environment (Friston, 2009, 2010). The FEP postulates that in order for an agent to exist (and persist) under time-varying environmental conditions, it must minimize a free energy functional under the agent's internal (“generative”) model for environmental observations (Friston et al., 2006).

Active inference, which is a corollary of the free energy principle, claims that natural agents act to fulfill prior beliefs about preferred observations (Friston, 2010). These prior beliefs about future observations are part of the agent's internal model specification, and free energy minimization thus ensures that the agent avoids surprising states.

Currently, the derivation of active inference algorithms on free-form dynamic models still requires manual work. Automation of active inference processes might enable practitioners to build more effective, flexible and scalable agents (de Vries and Friston, 2017). In addition, automated execution of active inference processes also requires the definition of a formal experimental protocol that governs the interaction between the agent and its environment.

The derivation of a free energy minimizing algorithm can be automated through the use of probabilistic programming (PP) techniques (Tran et al., 2016; Carpenter et al., 2017; Minka et al., 2018). While most PP toolboxes offer free-form modeling tools and automated derivation of flexible inference algorithms, their generality often comes at the cost of increased computational load. In contrast, dynamic models incorporate model-specific structure that can be exploited to increase algorithm performance. Here, message passing on a factor graph description of the generative model is especially suited for inference in flexibly definable dynamic models (Loeliger et al., 2007; Cox et al., 2019).

The current paper details an experimental protocol and simulation environment for the automated derivation and execution of online active inference processes in a dynamic context. Crucially, we illustrate how the message passing approach and proposed experimental protocol cooperate to automate the execution of structured active inference algorithms on flexibly definable generative dynamic models. We address the following questions:

1. How can online active inference processes be operationally described by an experimental protocol?
2. How can active inference processes be automatically derived within the given protocol?

With respect to the first issue, we describe a protocol that formally captures the interactions between an (active inference) agent and its environment. The protocol supports online simulations under situated conditions.

Concerning the second issue, the current paper provides a full message-passing based account of active inference, formulated in a Forney-style factor graph (FFG) representation of the internal model (Forney, 2001). The FFG formulation supports flexible model definitions and *automated* (active) inference execution by message passing-based free energy minimization. Crucially, this automation absolves the need for manual derivations of variational calculus problems and in principle scales up to complex hierarchical and interdependent models, making the approach suitable for industrial-sized applications.

The paper is structured as follows. Sections 2 and 3 offer a short technical rehearsal to active inference and FFGs, respectively. The experimental protocol is detailed in section 4. In section 5 we test our proposed protocol by simulating two classical active inference applications, namely the Bayesian thermostat and the mountain car. These simulations were executed with ForneyLab, which is a freely available toolbox for automated free energy minimization in FFGs that we have developed in our research lab (Cox et al., 2019). Finally, we discuss related work in section 6 and conclude in section 7.

## 2. Active Inference

**Figure 1:**
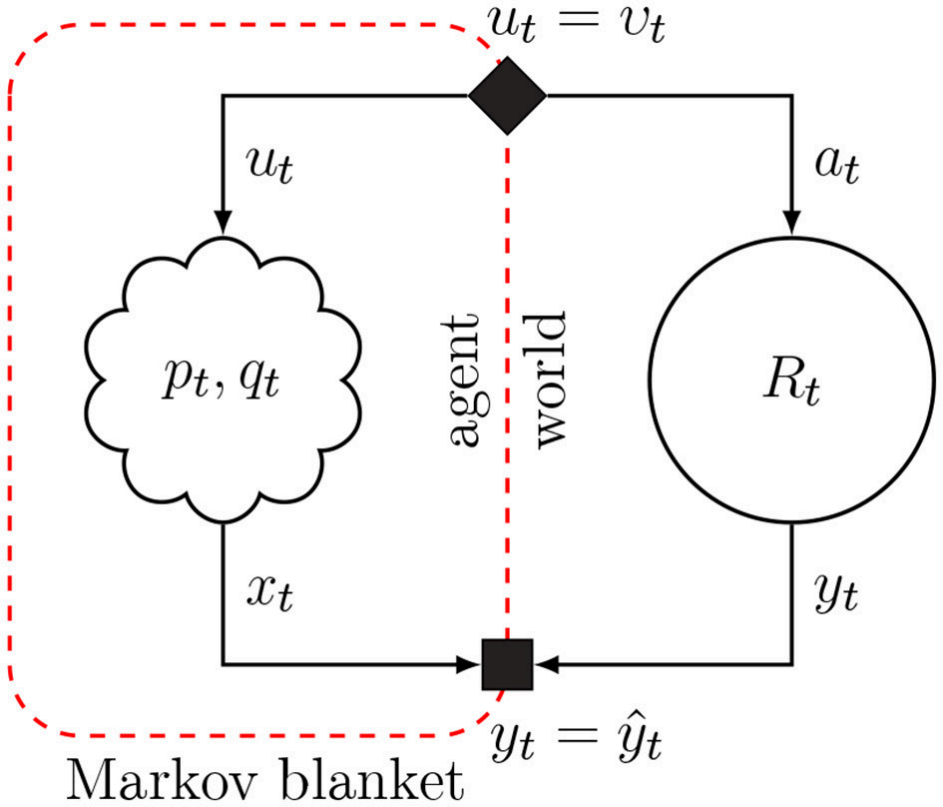
The Markov blanket forms a statistical separation between an agent and its environment. Arrows indicate the “generative” direction. Conditioning on the Markov blanket renders information about the internal states of the agent independent from information about hidden state of the environment. Blanket states for the agent are the variables for controls *u*_*t*_ and observations *x*_*t*_. The solid diamond node in the Markov blanket links posterior beliefs over the agent's control variables to environmental actions, and the solid square node in the Markov blanket passes environmental outcomes to agent observations.

Friston (2013) considers the consequences of being alive for the internal informational mechanics of natural agents. This analysis leads to the conclusion that natural agents appear to exchange information with their environment so as to maximize Bayesian evidence (i.e., minimize free energy) for an internal model of sensory data. We consider the system of interacting agent and environment as drawn in Figure 1. Both the agent and environment are considered dynamical systems with hidden state dynamics. The environment executes a process (*y*_*t*_, *z*_*t*_) = *R*_*t*_(*z*_*t*−1_, *a*_*t*_), where *a*_*t*_ is an action, *z*_*t*_ a latent state vector and *y*_*t*_ is the output signal. The agent holds a generative probabilistic model *p*_*t*_(*x, s, u*) for the environmental process, where *x*, *s*, and *u* are sequences of observations, internal states and controls. The action at time *t* in the environmental process is represented in the agent's model by control variable *u*_*t*_ and environmental output (*y*_*t*_) by sensory variable (“observation”) *x*_*t*_. The agent has a single goal, namely minimizing free energy, which roughly corresponds to minimizing (precision-weighted) prediction errors for its sensory inputs *x* (Rao and Ballard, 1999; Friston and Kiebel, 2009; Huang and Rao, 2011). Minimizing free energy leads to posterior beliefs over both the states *s* (which affects predictions and thereby prediction errors) and controls, which are observed by the environment as actions that lead to changes in the sensory inputs. These changes in sensory inputs may also affect prediction errors. Thus, inference for the states and controls both lead to free energy minimization. Technically, in order for a natural agent to persevere, it must be both physically and statistically separated from its environment. This statistical skin is called a *Markov Blanket*, which comprises the sensory and action variables. Sensory variables are affected by internal states of the environment but do not directly affect internal environmental states. Similarly, actions are affected by internal states of the agent but do not directly affect internal states of the agent.

Because active inference reasons from observations toward controls, the inference process requires the definition of an “inverse” probabilistic model which is sometimes called the *recognition model q*_*t*_. Full Bayesian inversion of *p*_*t*_ is in general intractable, so the agent resorts to approximating Bayesian inference by minimizing a variational free energy functional, defined by:

(1a) $$\begin{array}{rc} F\left[q_{t}\right] & =\int_{q_{t}}q_{t}\left(s,u\right)\log\frac{q_{t}\left(s,u\right)}{p_{t}\left(x,s,u\right)} \end{array}$$

(1b) $$\begin{array}{r} =\underset{\text{surprise}}{\underset{︸}{-\log p_{t}\left(x\right)}}+\underset{\text{posterior divergence}}{\underset{︸}{\int_{q_{t}}q_{t}\left(s,u\right)\log\frac{q_{t}\left(s,u\right)}{p_{t}\left(s,u|x\right)}}} \end{array}$$

Minimizing Equation (1) renders the free energy an upper bound on surprise (negative log-evidence), while simultaneously approximating the (generally unavailable) true posterior *p*_*t*_(*s, u*|*x*) with the recognition model *q*_*t*_. In order to render this optimization process tractable, the recognition model is often factorized (Attias, 1999), where a fully factorized recognition model is referred to as the “mean-field” assumption.

In order to equip the agent with a sense of “goal-directedness” or “purpose,” the internal model extends over future states and incorporates counter-factual beliefs about desired future outcomes, also referred to as *target priors* (Parr and Friston, 2018). These target priors lead to high surprisal for observations that are unlikely under the agent's preferences. Free energy minimization then produces (approximate) posterior beliefs over controls that are believed (by the agent) to avoid these undesired (surprising) observations. In the current paper we set the target priors ourselves, but more generally these priors might be set by contextual processes such as other agents or higher-level temporal layers. These ideas are further discussed in section 7.

Previous accounts of active inference introduce an expected free energy that steers goal-directed behavior via a prior over control (Friston et al., 2015; Friston K.J. et al., 2017). Instead, we will employ the internal model formulation by Parr and Friston (2018), which explicitly includes counter-factual prior beliefs over future observations in order to steer behavior. This leads to a unified model specification that allows us to optimize a single free energy functional (see also de Vries and Friston, 2017). Minimizing this functional by message passing simultaneously captures inference over current states (perception) as well as future controls (action/policy planning).

Practical models for active inference that are suitable for industrial application may be complex, layered and embedded in a volatile context. Manual derivation of free energy minimizing algorithms for active inference algorithms will then become prohibitively tedious and error-prone. A graphical representation of the internal model will aid with visualization of complex models and allows for automated derivation of message passing algorithms. The next section introduces Forney-style factor graphs as a graphical framework for automatic derivation of active inference algorithms.

## 3. Inference by Message Passing on Forney-style Factor Graphs

A Forney-style factor graph (FFG), also known as a “normal” factor graph, offers a graphical description of a factorized function (Forney, 2001). Excellent and detailed introductions to FFGs are available in (Loeliger, 2004; Korl, 2005). While related graphical formalisms such as Bayesian networks, Markov random fields and bipartite factor graphs offer essentially equivalent formulations (Forney, 2001; Loeliger, 2004), the FFG formalism is especially suited for representing dynamical models (Loeliger et al., 2007). Specifically, the FFG representation requires only a single node and message type, while retaining the explicit representation of variable relations through factor nodes.

As an example factorization, in this section we consider the function of Equation (2), which splits into four factors:

(2) $$\begin{array}{r} f\left(x_{1},x_{2},x_{3},x_{4}\right)=f_{a}\left(x_{1}\right)f_{b}\left(x_{1},x_{2},x_{4}\right)f_{c}\left(x_{2},x_{3}\right)f_{d}\left(x_{4}\right). \end{array}$$

**Figure 2:**
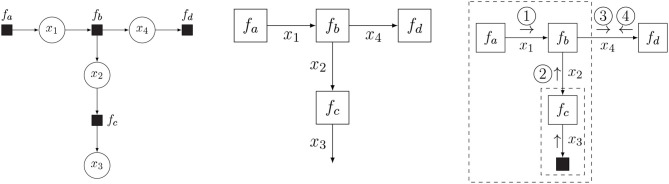
Bipartite factor graph **(left)** and Forney-style factor graph (FFG) **(middle)** for the model of Equation (2), together with the message passing schedule for computing (Equation 3b) **(right)**. In a bipartite factor graph, solid nodes represent factors and round nodes represent variables. In an FFG, edges represent variables and all nodes represent factors, where solid nodes indicate observations.

In this paper, we assume that the function *f* is a probability distribution. The FFG for this factorization is drawn in Figure 2 (middle), together with the equivalent bipartite factor graph representation (left) for comparison. In an FFG, variables correspond to edges and factors are represented by nodes. A node is connected to an edge only if the variable of the edge is an argument in the factor. For instance, node *f*_*c*_ connects to edges *x*_2_ and *x*_3_ since *f*_*c*_ = *f*_*c*_(*x*_2_, *x*_3_).

Now assume that we observe $x_{3}={\hat{x}}_{3}$ (technically: *x*_3_ is a variable that takes on value ${\hat{x}}_{3}$) and are interested in computing a posterior distribution for *x*_4_. The observation of *x*_3_ then imposes an additional constraint $\delta \left(x_{3}-{\hat{x}}_{3}\right)$ on the model, which clamps this variable to its observed value. Following (Reller, 2012), we indicate observations by a small solid node, see Figure 2 (right).

The integral for computing the posterior distribution is shown in Equation (3a). A direct approach to solving this integral might be tedious and error-prone. Conveniently, the distributive law allows us to pull unaffected integrands out of integrals, thus unpacking the total integral into a product of sub-integrals, each of which can be interpreted as a message over an edge of the factor graph, cf. Figure 2 (right) and Equation (3b):

(3a) $$\begin{array}{r} f\left(x_{4}|x_{3}={\hat{x}}_{3}\right)\\ \;\propto ∭f\left(x_{1},x_{2},x_{3},x_{4}\right)\delta \left(x_{3}-{\hat{x}}_{3}\right)\text{d}x_{1}\text{d}x_{2}\text{d}x_{3} \end{array}$$

(3b) $$\begin{array}{l} =\underset{③}{\underset{︸}{\iint \overset{①}{\overset{︷}{f_{a}(x_{1})}}\overset{②}{\overset{︷}{\int f_{c}(x_{2},x_{3})\delta (x_{3}-{\hat{x}}_{3})\text{d}x_{3}}}f_{b}(x_{1},x_{2},x_{4})\text{d}x_{1}\text{ d}x_{2}}}\\ \;\times \underset{④}{\underset{︸}{f_{d}(x_{4})}} \end{array}$$

(3c) $$={\vec{\mu }}_{③}(x_{4}){\overset{\leftarrow }{\mu }}_{④}(x_{4}).$$

Consecutive computation of these messages then leads to the solution of Equation (3c), where the (unnormalized) posterior function results from the product of the two colliding messages ③ and ④.

For efficiency reasons the message normalizing constants are often ignored, and messages are represented by a probability distribution with corresponding sufficient statistics. The message can then be interpreted as an information summary for the variable of the corresponding edge. For instance, message ③ in Figure 2 (right) represents the probability distribution for *x*_4_ given the information in the large box at left-hand side of message ③. While an FFG is principally an undirected graph, we often draw arrows on the edges in order to anchor the message direction. A forward message $\vec{\mu }$ aligns with the edge arrow, and a backward message $\overset{⃖}{\mu }$ aligns against the edge direction.

Crucially, the message passing approach to inference allows to reuse pre-derived solutions to specific message updates for elementary factors across multiple models. Implementing these solutions in a look-up table allows us to automate the derivation and execution of message passing algorithms. Well-known algorithms such as (loopy) belief propagation (Forney, 2001), variational message passing (Dauwels, 2007), expectation maximization (Dauwels et al., 2005), and expectation propagation (Cox, 2018) have all been formulated as message passing procedures on an FFG.

### 3.1. Example: Equality Node

In order to exemplify the derivation of a reusable message update rule for an elementary factor, we consider the equality factor (see also Korl, 2005; Cox et al., 2019), defined as

(4) $$\begin{array}{r} f_{=}\left(x,y,z\right)=\delta \left(z-x\right)\delta \left(z-y\right). \end{array}$$

In contrast to a variable in a bipartite graph (Figure 2, left), a variable in an FFG can connect to at most two factors. The equality factor resolves this situation by constraining the information about three variables to be equal (Equation 4). Edges constrained by equality factors can then effectively be regarded as a single variable that is shared among connected factors.

**Figure 3:**
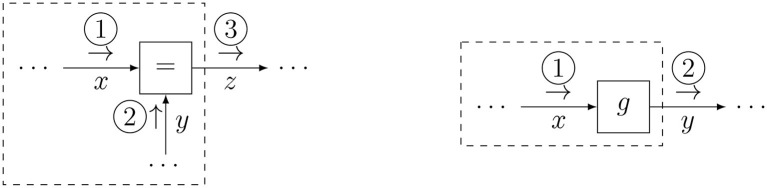
Message updates for the equality node **(left)** and the nonlinear node **(right)**.

An update for the equality node is computed by Equation (5b), for which the schedule is graphically represented by Figure 3 (left). It can be seen that this schedule fuses information from two branches into a single message, much like Bayes rule does. Indeed, in FFGs, equality nodes are often used to connect prior information about a variable with likelihood-based information about that variable.

(5a) $${\vec{\mu }}_{③}(z)=\iint {\vec{\mu }}_{①}(x){\vec{\mu }}_{②}(y)f_{=}(x,y,z)\text{ d}x\text{ d}y$$

(5b) $$={\vec{\mu }}_{①}(z){\vec{\mu }}_{②}(z).$$

### 3.2. Dealing With Nonlinear Factors

The modularity of the message passing approach allows to make local approximations. Through local linearization, we can pass messages through nodes that encode nonlinear constraints. Here we consider the factor of Equation (6), where *g*(*x*) is a nonlinear differentiable function:

(6) $$\begin{array}{r} f_{g}\left(x,y\right)=\delta \left(y-g\left(x\right)\right). \end{array}$$

The forward message is expressed as Equation (7a) and drawn in Figure 3 (right).

(7a) $$\begin{array}{c} {\vec{\mu }}_{②}(y)=\int {\vec{\mu }}_{①}(x)f_{g}(x,y)\text{ d}x \end{array}$$

(7b) $$\begin{array}{c} ={\vec{\mu }}_{①}(g^{-1}(y)). \end{array}$$

When the incoming message ① is in the exponential family, it can be conveniently parameterized by its sufficient statistics. However, because of the nonlinear transformation, the outgoing message is no longer guaranteed to be a member of the exponential family, which may lead to intractable updates. Therefore, we trade some accuracy for computability by making a local linear approximation to the node function. We choose the approximation point $\hat{x}$ as the mean of the incoming message, and expand *g* locally as

(8) $$\begin{array}{rc} \tilde{g}\left(x\right) & =g\left(\hat{x}\right)+J_{g}\left(\hat{x}\right)\left(x-\hat{x}\right), \end{array}$$

where $J_{g}\left(\hat{x}\right)$ is the Jacobi matrix at the approximation point (or the first derivative in the scalar case). By substituting Equation (8) in Equation (6), we can obtain approximate but tractable message updates. This local linearization strategy for nonlinear factors will be used in section 5.2.

## 4. Online Active Inference in Factor Graphs

In section 2 we mentioned the agent's internal model *p*_*t*_, which expresses the agent's prior beliefs about how the environmental process generates observations from actions. In the current section we propose a simulation protocol for online active inference execution by the agent.

### 4.1. Model Specification

**Figure 4:**
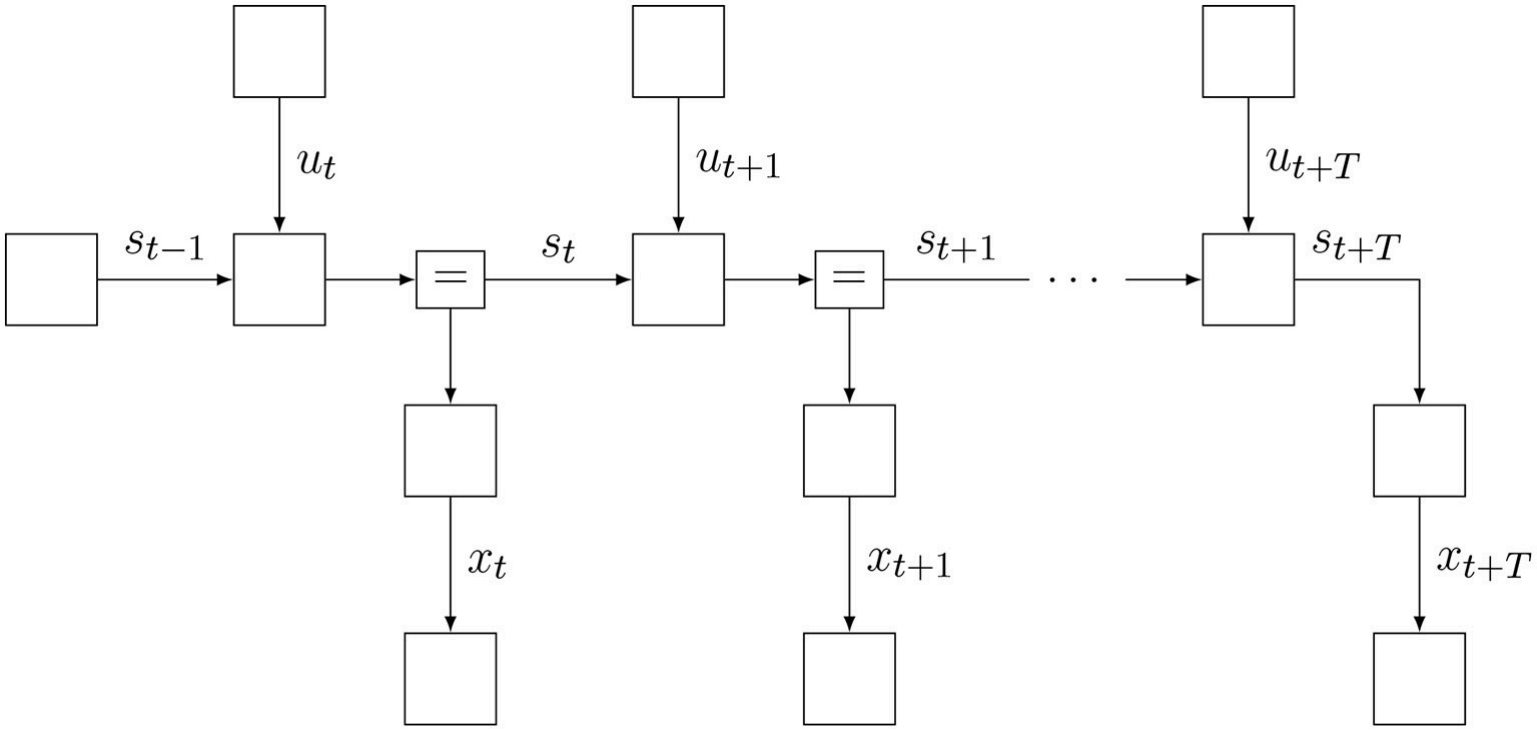
The Forney-style factor graph for the agent's generative model (Equation 9).

We consider an agent with a state-space model (SSM) factorization for its internal model, given by

(9) $$\begin{array}{r} p_{t}\left(x,s,u\right)\propto p\left(s_{t-1}\right)\overset{t+T}{\underset{k=t}{\prod }}\underset{\text{observation}}{\underset{︸}{p\left(x_{k}|s_{k}\right)}}\underset{\text{state transition}}{\underset{︸}{p\left(s_{k}|s_{k-1},u_{k}\right)}}\underset{\text{control}}{\underset{︸}{p\left(u_{k}\right)}}\underset{\text{target}}{\underset{︸}{p^{\prime }\left(x_{k}\right)}}. \end{array}$$

where *x*, *s*, and *u* are sequences with ranges that are implicitly given by the model specification at the right-hand side. Note how the model of Equation (9) differs slightly from a standard SSM factorization (Koller and Friedman, 2009), because it includes additional “target” priors $p^{\prime }\left(x_{k}\right)$ over desired future outcomes. A factor graph representation of Equation (9) is shown in Figure 4. Also note that the probability distribution for the internal model has a subscript *t* to indicate that the model is time-varying. We will consider these aspects in more detail below.

Also note that at time step *t*, the agent has assumptions about how the environment will evolve over the next *T* + 1 time steps since we can run this model forward and generate observations *x*_*k*_ for *k* = *t, t* + 1, …, *t* + *T*. The horizon *T* is determined by the information content of the target priors $p^{\prime }\left(x_{k}\right)$. These target priors are generally set by states of contextual processes, i.e., not by this agent but rather by other agents (or higher level processes) that encode unsurprising future outcomes for this agent. In order to distinguish the predictive model for observations *p*(*x*_*k*_|*s*_*k*_) from the context-based target prior for observations $p^{\prime }\left(x_{k}\right)$, we label the latter factor with a prime.

We refer to a sequence of future controls *u* = (*u*_*t*_, *u*_*t* + 1_, …, *u*_*t* + *T*_) as a *policy*. Through inference, the posterior over policies becomes dependent on the hidden state sequence *s*. Prior to inference however, the model requires the definition of a prior belief over policies that constrains attainable control states. In a more general formulation of the internal model, we would write a prior over policies *p*(*u*) = *p*(*u*_*t*_, *u*_*t* + 1_, …, *u*_*t* + *T*_). Here, for simplicity, we assume $p\left(u\right)=\prod_{k=t}^{t+T}p\left(u_{k}\right)$.

Next to the internal model, we assume that the agent has access to a variational distribution, also known as the *recognition* distribution,

(10) $$\begin{array}{r} q_{t}\left(x,s,u\right) \end{array}$$

that encodes the agent's posterior beliefs about all latent states. Because the future is by definition unobserved, the recognition distribution includes the future observation variables as well. The distinction between the agent's prior (“generative”) beliefs *p*_*t*_(*x, s, u*) and posterior (“recognition”) beliefs *q*_*t*_(*x, s, u*) will be finessed below. At the start of the simulation (say at *t* = 1), we will set *q*_1_ to an uninformative distribution. As time progresses, observations become available and subsequent inference iterations will tune *q*_*t*_ to more informed distributions. Often, but not necessarily so, the recognition distribution is assumed to be fully factorized (this is the *mean-field* assumption). In the present article we assume a structured factorization for the recognition distribution that is solely induced by the internal model factorization (Bishop, 2006). In general, we may write

$$\begin{array}{ll} q_{t}\left(x,s,u\right) & =q\left(x,s|u\right)q\left(u\right). \end{array}$$

Since actions onto the environment are real-valued interventions, we will generally enforce a deterministic posterior belief over policies, i.e.,

(11) $$\begin{array}{r} q\left(u_{t},\ldots ,u_{t+T}\right)=\overset{t+T}{\underset{k=t}{\prod }}\delta \left(u_{k}-\upsilon_{k}\right), \end{array}$$

where υ_*k*_ (upsilon) are parameters that are to be determined in the free-energy minimization process (see section 4.3). In order words, while the prior belief over policies *p*(*u*) may contain uncertainties, we will fix the posterior belief over policies *q*(*u*) on a single concrete sequence.

As time progresses, at each time step, the agent interacts with its environment through exchanging actions and observations, followed by processing the observations. Technically, everything that the agents does can be described as updates to its internal and recognition models. We distinguish three phases per time step: (1) act-execute-observe, (2) infer (process observations), and (3) slide (move one time slice ahead), see Algorithm 1. Next, we consider each step in more detail.

**Algorithm 1:** The online active inference algorithm

**Require:** *p*_1_(*x, s, u*) and *q*_1_(*x, s, u*) //*model specification*
1: **for** *t* = 1 to ∞ **do**
2: **Act-Execute-Observe** // *update to p*_*t*_(*x, s, u*)
3: **Infer** // *update to q*_*t*_(*x, s, u*)
4: **Slide** // *move one time slice ahead*
5: **end for**

### 4.2. The “Act-Execute-Observe” Step

Since we assumed that the posterior beliefs over control states were constrained by δ(*u*_*k*_ − υ_*k*_), we will select the *action* at time step *t* as

(12) $$\begin{array}{r} a_{t}=\upsilon_{t}. \end{array}$$

Technically, in a factor graph context, we will let the agent be the owner of the control state *u*_*t*_ and the environment is the owner of action variable *a*_*t*_. The agent and environment are coupled at the agent's Markov blanket by an interface factor δ(*u*_*t*_ − *a*_*t*_). Message passing from the agent to the environment will now pass the agent's posterior belief *q*(*u*_*t*_) = δ(*u*_*t*_ − υ_*t*_) over control state *u*_*t*_ to the action, leading to *a*_*t*_ = υ_*t*_. Next, this action is imposed onto the environmental process *R*_*t*_ that generates outputs *y*_*t*_ by

(13) $$\begin{array}{r} \left(y_{t},z_{t}\right)=R_{t}\left(z_{t-1},a_{t}\right), \end{array}$$

where *z*_*t*_ refers to the dynamic states of the environmental process. Here, we call environmental processing the *execution* phase. In similar fashion to the action-interface, if we let *y*_*t*_ refer to the output *variable* and ŷ_*t*_ is the *value* of the environmental output at time *t*, then an observation-interface node δ(*x*_*t*_ − *y*_*t*_) at the agent's Markov blanket leads to an incoming message δ(*x*_*t*_ − ŷ_*t*_) from observation variable *x*_*t*_. In a real-world setting, the agent does not know the model *R*_*t*_ nor the environmental states, but still gets to observe the environmental output. Acting onto the environment and observing the consequences can technically be processed by the agent through updating its internal model to

(14) $$\begin{array}{r} p_{t}\left(x,s,u\right):=p_{t}\left(x,s,u\right)\underset{\text{action and observation}}{\underset{︸}{\delta \left(u_{t}-\upsilon_{t}\right)\delta \left(x_{t}-{ŷ}_{t}\right)}}. \end{array}$$

**Figure 5:**
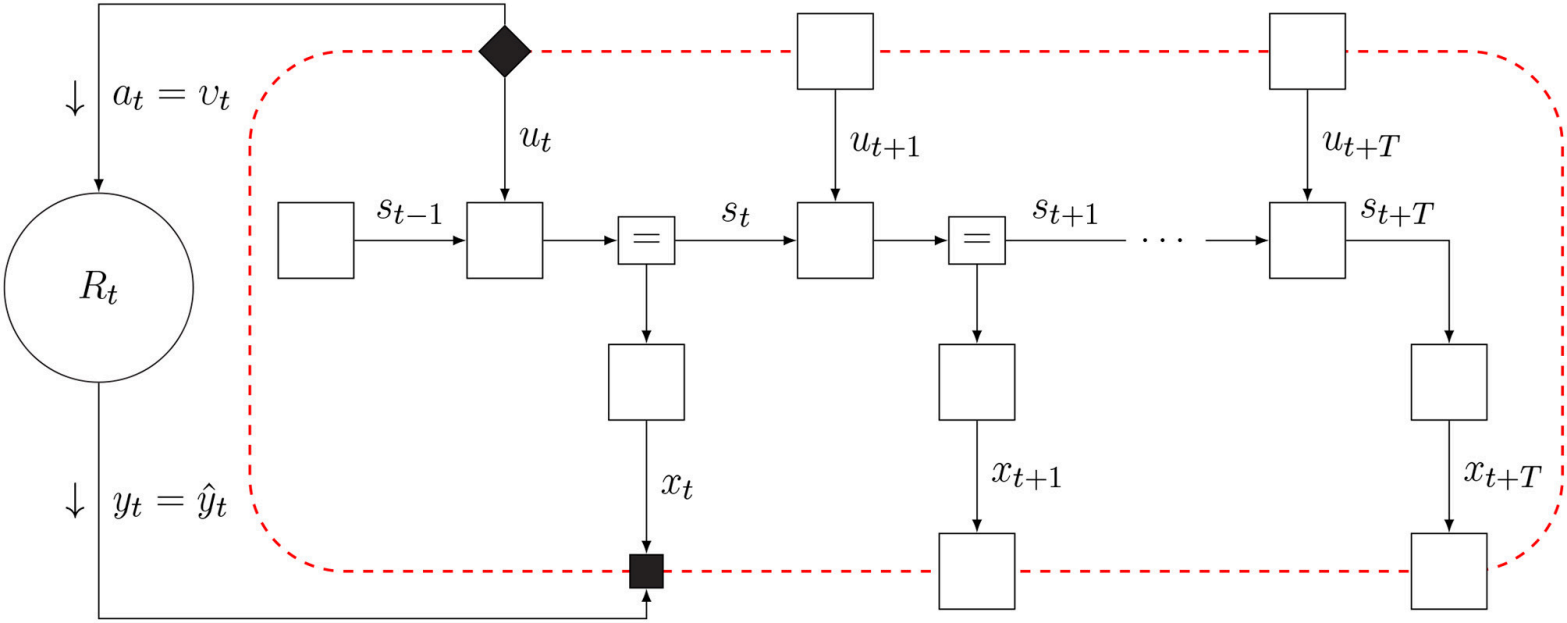
The FFG for the act-execute-observe phase.

The factor graph of the updated internal model is shown in Figure 5. We use small filled diamond nodes to indicate causal interventions (actions) and small square nodes for observation variables. Note that when we multiply the model with a delta distribution δ(*u*_*t*_ − υ_*t*_), we technically overwrite the prior *p*(*u*_*t*_) and the open square for *p*(*u*_*t*_) is replaced by a black diamond smaller square to indicate the intervention. Similarly, the target prior $p^{\prime }\left(x_{t}\right)$ is omitted because the observation δ(*x*_*t*_ − ŷ_*t*_) renders it conditionally independent from the rest of the model.

### 4.3. The “Infer” Step

The internal model has changed as a result of acting and observing. In the infer step, we process the consequences of this change for the model's latent variables. Technically, we update the posterior from *q*_*t*−1_ to *q*_*t*_ by free energy minimization. Generally, the recognition distribution will be parameterized by sufficient statistics μ, so we can write *q*_*t*_(*x, s, u*) = *q*(*x, s, u*|μ_*t*_) and free energy minimization amounts to finding μ_*t*_ by

(15) $$\begin{array}{rc} \mu_{t} & =\arg\underset{\mu }{\min}\underset{\text{free energy}F_{t}\left(\mu \right)\text{at time step}t}{\underset{︸}{\int q\left(x,s,u|\mu \right)\log\frac{q\left(x,s,u|\mu \right)}{p_{t}\left(x,s,u\right)}\text{d}x\text{d}s\text{d}u}} \end{array}$$

**Figure 6:**
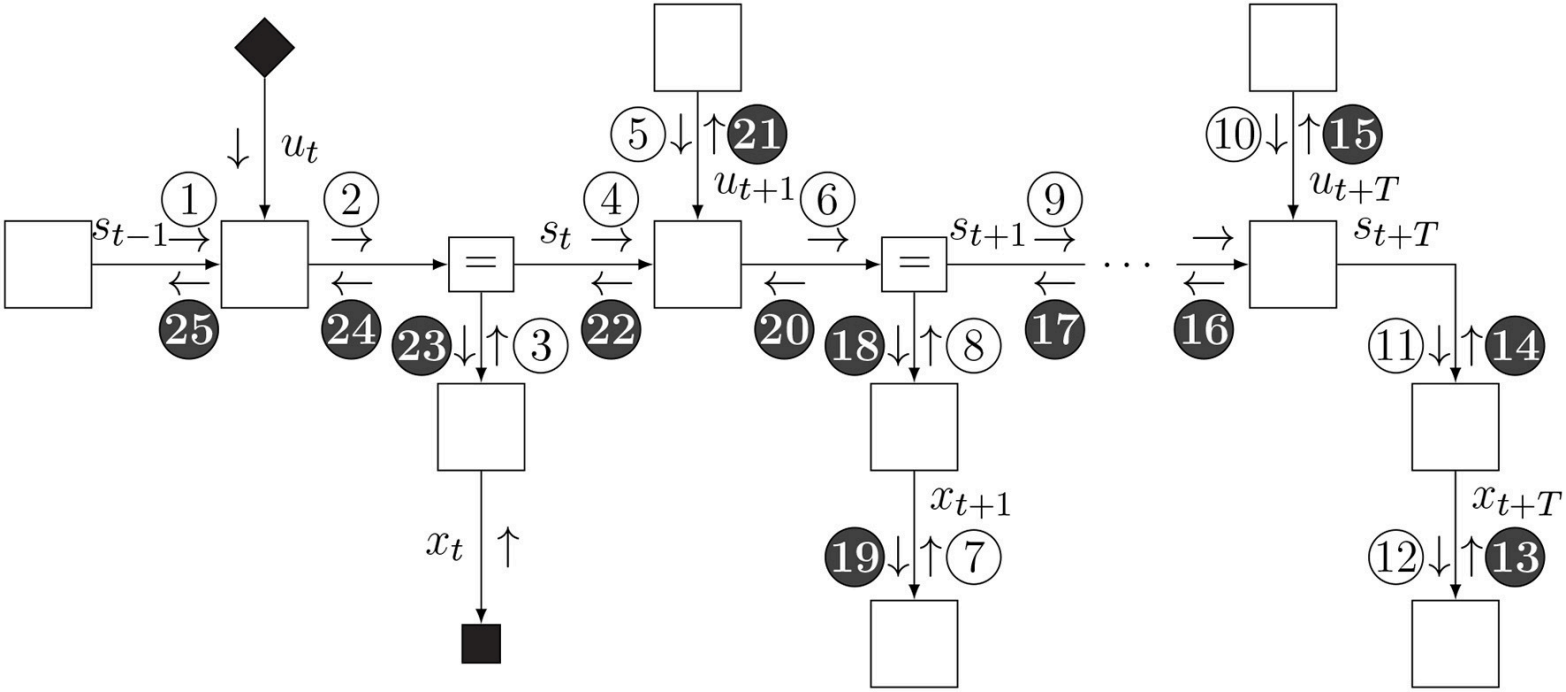
Schedule for infer phase. Light messages constitute a forward (filtering) pass, and dark messages constitute a backward (smoothing) pass.

This minimization procedure can be executed through variational message passing (VMP) in the factor graph for the updated internal model *p*_*t*_(*x, s, u*). The inference process is visualized in Figure 6. VMP involves iteratively updating single (or a few) components of μ while holding the other components fixed. In this procedure, it will be very useful to set the initial value $\mu_{t}^{\text{init}}$ to μ_*t*−1_. With this initialization, in most cases, the number of VMP iterations to convergence per time step will be quite low.

### 4.4. The “Slide” Step

**Figure 7:**
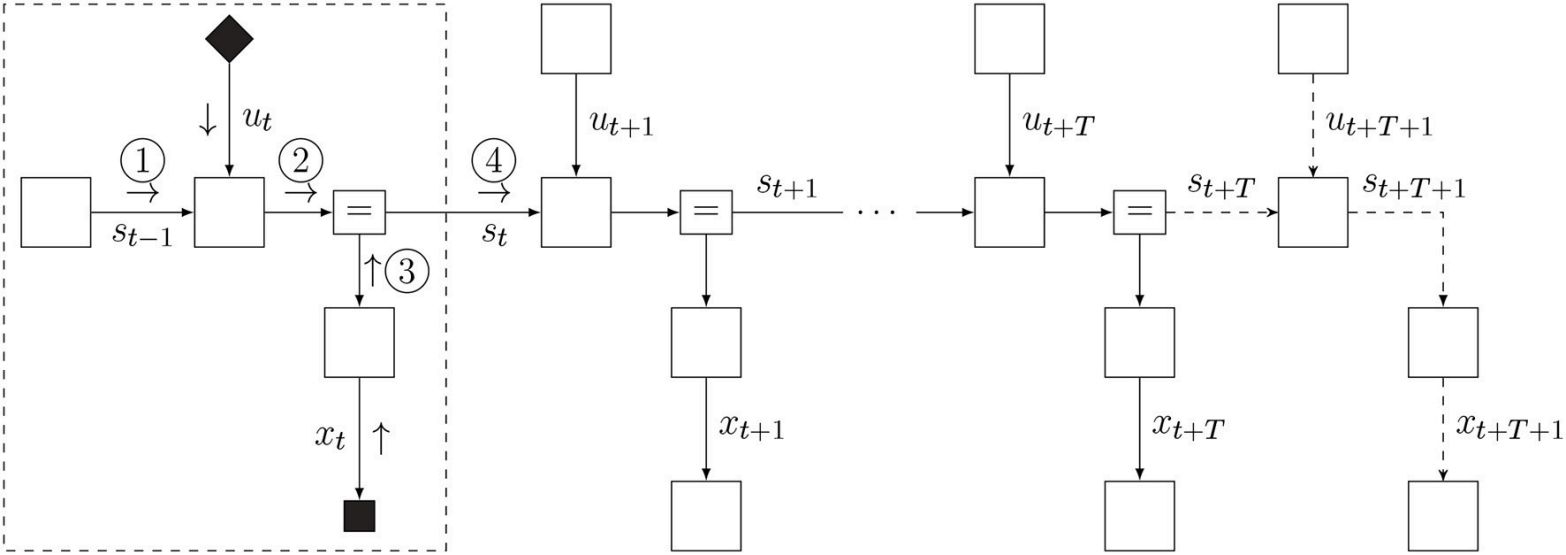
Schedule and FFG extension for slide phase.

The slide step implements a time step increment and is best understood by looking at the factor graph in Figure 7. Essentially, we eliminate (marginalize out) the first time slice of the internal and recognition models and add a time slice to the horizon. Then we fix the indexing back to *p*_*t*_.

Formally, marginalization of the first slice and adding a slice at the horizon leads to the following update of the agent's internal model:

(16) $$\begin{array}{rc} p_{t+1}\left(x,s,u\right)\propto & \underset{\text{close box (marginalization) for first slice, yielding new prior}p\left(s_{t}\right)}{\underset{︸}{\int \overset{\text{first slice}}{\overset{︷}{p\left(s_{t-1}\right)p\left(s_{t}|s_{t-1},u_{t}\right)p\left(x_{t}|s_{t}\right)}}\overset{\text{action and observation}}{\overset{︷}{\delta \left(u_{t}-a_{t}\right)\delta \left(x_{t}-y_{t}\right)}}\text{d}s_{t-1}\text{d}u_{t}\text{d}x_{t}}}\cdot \\ \underset{\text{unaltered mid-section slices}}{\underset{︸}{\left(\overset{t+T}{\underset{k=t+1}{\prod }}p\left(x_{k}|s_{k}\right)p\left(s_{k}|s_{k-1},u_{k}\right)p\left(u_{k}\right)p^{\prime }\left(x_{k}\right)\right)}}\cdot \\ \underset{\text{add slice at horizon}}{\underset{︸}{p\left(x_{t+T+1}|s_{t+T+1}\right)p\left(s_{t+T+1}|s_{t+T},u_{t+T+1}\right)p\left(u_{t+T+1}\right)p^{\prime }\left(x_{t+T+1}\right)}} \end{array}$$

Here, we have processed the action and observation together with sliding a time step in one update. The marginalization of *x*_*t*_, *s*_*t*−1_, and *u*_*t*_ in the first slice of the graph can be executed by message passing. In practice, we can simply re-use message ④ from Figure 6 (after re-normalization) as the new state prior *p*(*s*_*t*_). In order to maintain a horizon of *T* time steps, we add one time slice of the state space model to the horizon (at time step *t* + *T* + 1). Again, it is assumed that a contextual agent feeds the target prior $p^{\prime }\left(x_{t+T+1}\right)$.

Adding and deleting slices at each time step also provides room for changing the model structure of the internal model on the basis of current contextual information. For instance, the observation model *p*(*x*_*t* + *T* + 1_|*s*_*t* + *T* + 1_) does not need to be the same as *p*(*x*_*t* + *T*_|*s*_*t* + *T*_). The decision for the structure of *p*(*x*_*t* + *T* + 1_|*s*_*t* + *T* + 1_) can be postponed until time step *t*.

Because the recognition model is simply parameterized by its sufficient statistics [*q*_*t*_(*x, s, u*) = *q*(*x, s, u*|μ_*t*_)], we do not require an explicit slide for the recognition model. In the next “infer” step, the recognition model is simply initialized with the present statistics ($\mu_{t}^{init}=\mu_{t-1}$) and updated to the new posterior statistics, without the need for redefining the recognition model.

Once the internal model has slided forward by one slice, we increment the time step index by

(17) $$\begin{array}{r} t:=t+1 \end{array}$$

so as to obtain a generative model *p*_*t*_(*x, s, u*) again (see Equation 9), but now for an increased time step. The Slide phase is followed by repeating the loop, starting with an Act-Execute-Observe step.

In summary, online active inference by a dynamic agent proceeds according to updating both its internal and recognition distributions at each time step according to Equations 9, 11, 15, 16, and 17. Actions and observations are technically processed by appending delta factors to the agent's internal model. The effects of these internal model changes on the latent variables in the model are inferred through free energy minimization, which leads to an updated recognition distribution. Next, the model slides forward one time slice and starts a new Act-Execute-Observe step.

## 5. Simulations

In order to illustrate the operationability of the active inference protocol, we simulate two classic active inference problems, namely the Bayesian thermostat (Friston et al., 2012; Buckley et al., 2017) and the mountain car (Friston et al., 2009; Sutton and Barto, 2018; Ueltzhöffer, 2018). We use ForneyLab (version 0.9.1) as a tool for automated derivation of message passing algorithms (Cox et al., 2019). ForneyLab (available from https://github.com/biaslab/ForneyLab.jl) supports flexible specifications of factorized probabilistic dynamic models (van de Laar et al., 2018) and generates high-performance inference algorithms on these models (Cox et al., 2019).

ForneyLab is written in Julia, a high-level programming language for numerical computing. Julia combines an accessible MATLAB-like syntax with C-like performance (Bezanson et al., 2017). Julia's excellent meta-programming capabilities allow ForneyLab to define an intuitive domain-specific model specification syntax, and to automatically compile message passing schedules directly to executable Julia code. Furthermore, Julia's powerful multiple dispatch functionality enables efficient scheduling and algorithm execution.

### 5.1. The Bayesian Thermostat

In this section we simulate an agent that can relocate itself in a temperature gradient field. The agent aims to position itself at a desired temperature relative to a heat source. Our simulation setup is an adaptation of the setup described by Buckley et al. (2017).

#### 5.1.1. Environmental Process Specification

**Figure 8:**
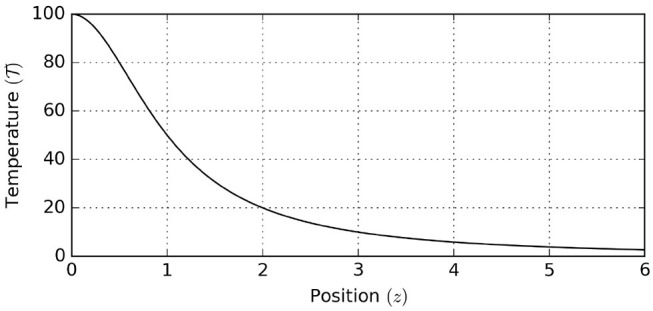
Environmental temperature profile, showing environmental temperature as a function of position.

First we specify the environmental process. The temperature T as a function of position *z* follows the profile

(18) $$\begin{array}{r} T\left(z\right)=\frac{T_{0}}{z^{2}+1}, \end{array}$$

where $T_{0}=100$ represents the temperature at the location of the heat source, see Figure 8.

The environmental process is steered by actions *a*_*t*_ that reflect the velocity of the agent. The output of the environmental process is the observed temperature by the agent. We assume that the agent observes a noisy temperature, leading to the following environmental process equations:

(19a) $$\begin{array}{rc} z_{t} & =z_{t-1}+a_{t} \end{array}$$

(19b) $$\begin{array}{rc} y_{t} & \sim N\left(T\left(z_{t}\right),\vartheta \right). \end{array}$$

In our simulation, we used initial position *z*_0_ = 2 and observation noise variance ϑ = 10^−2^.

#### 5.1.2. Internal Model Specification

The agent's internal model follows the factorization of Equation (9). The goal prior encodes a belief about a preferred temperature *x*_
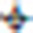
_ = 4, as 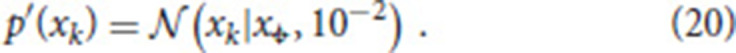


Furthermore, we endow the agent with an accurate model of system dynamics

(21) $$\begin{array}{r} p\left(s_{k}|s_{k-1},u_{k}\right)=N\left(s_{k}|s_{k-1}+u_{k},10^{-2}\right). \end{array}$$

However, in order to challenge the agent, we hamper the observation model. Instead of the actual temperature profile (Equation 19b), we use

(22) $$\begin{array}{r} p\left(x_{k}|s_{k}\right)=N\left(x_{k}|-s_{k},10^{-2}\right), \end{array}$$

which simply specifies that the observed temperature decreases with position. The internal model definition is completed by specifying a prior for controls and a vague prior for the initial state:

(23a) $$\begin{array}{rc} p\left(u_{k}\right) & =N\left(u_{k}|0,10^{-2}\right) \end{array}$$

(23b) $$\begin{array}{rc} p\left(s_{0}\right) & =N\left(s_{0}|0,10^{12}\right). \end{array}$$

**Figure 9:**
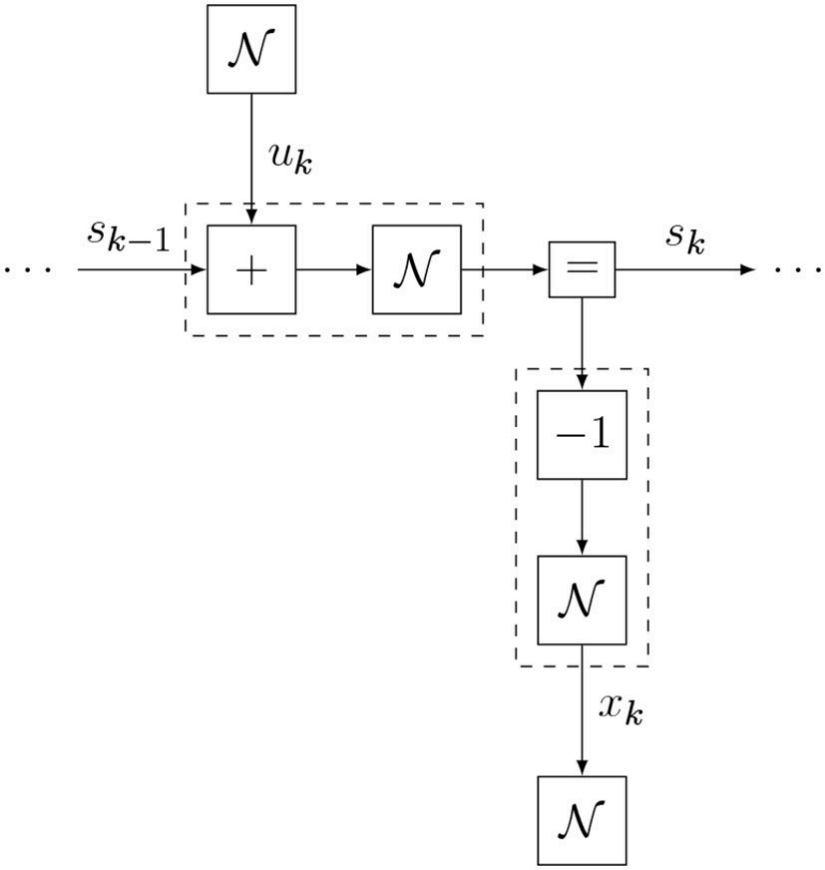
One time section of the Bayesian thermostat model (excluding the state prior).

**Figure 10:**
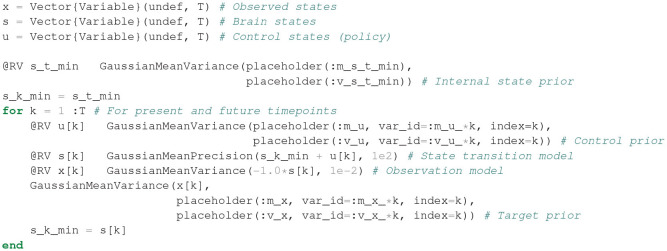
Julia code for building the internal model for the Bayesian thermostat with ForneyLab. In Julia, the prefix “@” indicates a macro. Under the hood, @RV constructs the Forney-style factor graph for the specified random variables. The “:” prefix (e.g., :m_x) specifies a symbol that may be used for indexing. placeholder indicates a placeholder for data that can be passed to the algorithm during inference.

Substituting Equations (20–23) in Equation (9) then returns the full internal model definition. One section of the generative model FFG is shown in Figure 9. A Julia code snippet^1^ for constructing the internal model with ForneyLab is shown in Figure 10.

**Figure 11:**
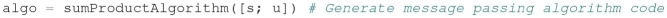
ForneyLab command for automated generation of the message passing algorithm for inference in the Bayesian thermostat simulation. The sum-product algorithm implicitly assumes the recognition model of Equation (11). ForneyLab also supports generating message passing code for alternative constraints on the recognition distribution (see e.g., Cox et al., 2018).

**Figure 12:**
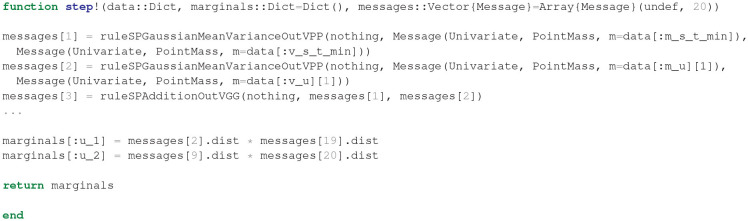
Snippet of the automatically generated inference algorithm code (*T* = 2) for the Bayesian thermostat. Upon inference, the step! function builds an array of messages using pre-derived update rules (e.g., ruleSPAdditionOutVGG) and returns posterior beliefs over internal and control states.

After having specified the generative model, ForneyLab can be used to automatically generate a message passing algorithm for free energy minimization, see Figure 11. ForneyLab generates Julia source code for the message passing algorithm, see Figure 12 for a snippet of the generated code.

#### 5.1.3. Simulation Results

**Figure 13:**
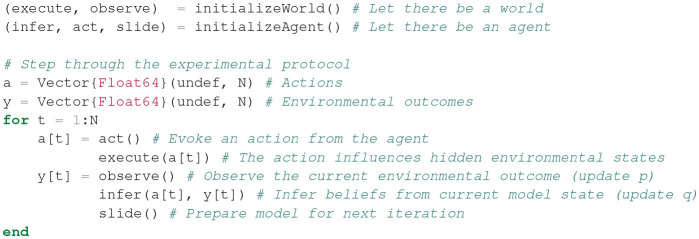
Code for executing the experimental protocol. The initializeWorld and initializeAgent functions specify closures that return functions for interacting with the environment and agent. This construct allows for encapsulating hidden states in respective scopes for the agent and environment, and allowing only indirect access to environmental states through returned functions.

The agent-environment system was simulated using the experimental protocol as defined in section 4, for 100 time steps and with horizon *T* = 2, with the additional constraint that the agent is only allowed to act after *t* = 25. Execution of the experimental protocol amounts to executing the code of Figure 13. Note that this implementation of the experimental protocol ensures that the agent never observes the environmental states of the environment directly, but rather only interacts with the environmental process through the act, execute, and observe functions. This setup emphasizes the idea that the environmental states (*z*) are not (directly) observable. In principle, this setup is then applicable to a real-world setting where the environmental process is not a simulation.

**Figure 14:**
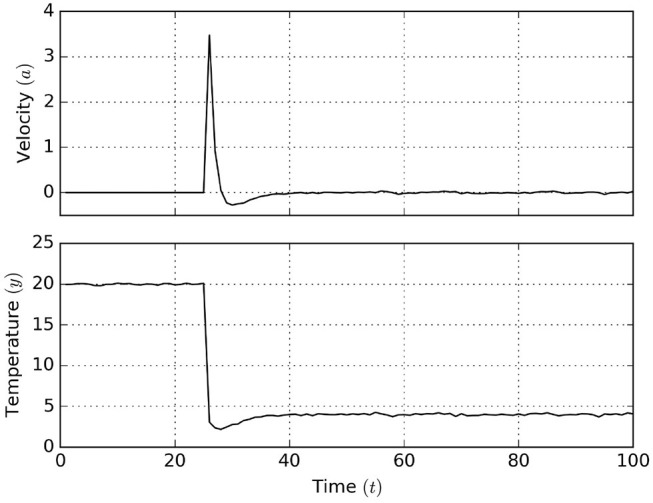
Active inference results for the Bayesian thermostat. After *t* = 25 the agent is allowed to act, moving toward the goal temperature *x*_
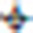
_ = 4.

The graphs of Figure 14 show the simulation results. It can be seen that after the agent is allowed to act, it quickly moves away from the heat source in order to eventually settle at the desired temperature. Apparently, despite the hampered observation model, the agent still attains the desired goal.

### 5.2. Mountain Car

In this section we simulate an agent that aims to relocate and park itself on a steep hill. However, the agent's engine is too weak to climb the hill directly. Therefore, a successful agent should first climb a neighboring hill, and subsequently use its momentum to overcome the steep incline on the goal-side. This task is also known as the mountain car problem (Friston et al., 2009), which is considered a classical benchmark in the reinforcement learning literature (Sutton and Barto, 2018).

#### 5.2.1. Environmental Process Specification

**Figure 15:**
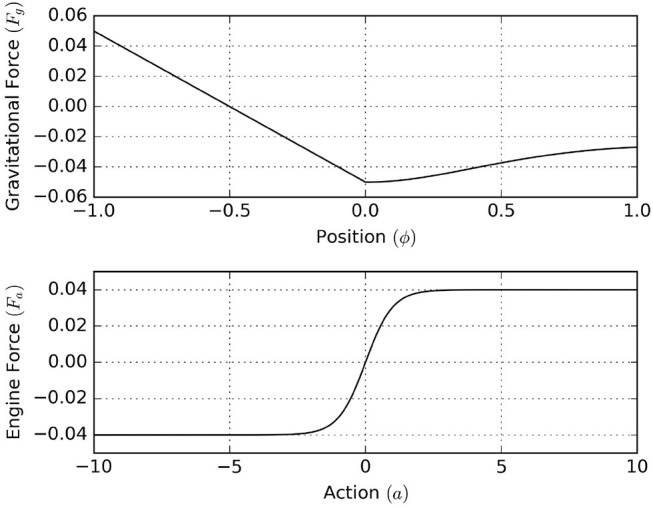
**(Top)** The horizontal gravitational force component that acts upon the agent as a function of position. **(Bottom)** The horizontal force component of the engine as a function of action.

We start by defining the environmental process, which is similar to the process defined in Ueltzhöffer (2018). We interpret the environmental state $z_{t}=\left(\phi_{t},{\overset{\cdot }{\phi }}_{t}\right)$, as the respective position and velocity of the mountain car. The horizontal gravitational force component of the hilly landscape depends upon the mountain car's position by [see Figure 15 (top)]

(24) $$\begin{array}{ll} F_{g}\left(\phi \right) & =\{\begin{array}{cc} -0.05\left(2\phi +1\right) & \text{if}\phi <0\\ -0.05\left[{\left(1+5\phi^{2}\right)}^{-1/2}+\phi^{2}{\left(1+5\phi^{2}\right)}^{-3/2}+\frac{1}{16}\phi^{4}\right] & \text{otherwise}, \end{array} \end{array}$$

Furthermore, we define a velocity-dependent drag as

(25) $$\begin{array}{rc} F_{f}\left(\overset{\cdot }{\phi }\right) & =-0.1\overset{\cdot }{\phi }. \end{array}$$

Through actions, the agent is allowed to set the engine force, which is limited to the interval (−0.04, 0.04), by [see Figure 15 (bottom)]

(26) $$\begin{array}{rc} F_{a}\left(a\right) & =0.04\tanh\left(a\right). \end{array}$$

Assuming unit mass for the mountain car, this leads to the following discrete system dynamics:

(27a) $$\begin{array}{rc} {\overset{\cdot }{\phi }}_{t} & ={\overset{\cdot }{\phi }}_{t-1}+F_{g}\left(\phi_{t-1}\right)+F_{f}\left({\overset{\cdot }{\phi }}_{t-1}\right)+F_{a}\left(a_{t}\right) \end{array}$$

(27b) $$\begin{array}{rc} \phi_{t} & =\phi_{t-1}+{\overset{\cdot }{\phi }}_{t}. \end{array}$$

In our simulation, we choose initial position ϕ_0_ = −0.5 and initial velocity ${\overset{\cdot }{\phi }}_{0}=0$. The environmental process generates outcomes as noisy observations of the current state with an observation noise variance θ = 10^−4^·*I*, where *I* represents the 2 × 2 identity matrix. This leads to the following environmental system dynamics:

(28a) $$\begin{array}{rc} z_{t} & =g\left(z_{t-1},a_{t}\right) \end{array}$$

(28b) $$\begin{array}{rc} y_{t} & \sim N\left(z_{t},\theta \right), \end{array}$$

where Equations (27a, 27b) are summarized by a transition function *g*(·).

#### 5.2.2. Internal Model Specification

For the internal model we define observation variables $x_{k}=\left(\xi_{k},{\overset{\cdot }{\xi }}_{k}\right)$, and encode the agent's target to reach a desired state *x*_
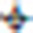
_ = (ξ_
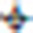
_, $\dot{\xi }$_
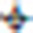
_) = (0.5, 0) at time *t* = 20 by defining a time-dependent target prior 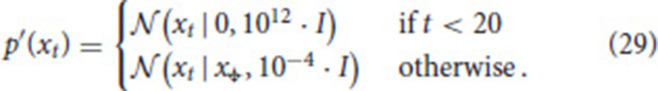


In other words, we set a vague prior belief over short-term observations (*t* < 20), but aim to reach and remain at the goal state afterwards.

We endow the agent with an accurate model of the environmental dynamics. These dynamics are captured by the transition model for the internal states $s_{k}=\left(\zeta_{k},{\overset{\cdot }{\zeta }}_{k}\right)$. Because the current state is a non-linear function of the previous state and action, we resort to local linear approximations of the system dynamics as explained in section 3.2. We choose the transition model

(30) $$\begin{array}{r} p\left(s_{k}|s_{k-1},u_{k}\right)=N\left(s_{k}|\tilde{g}\left(s_{k-1},u_{k}\right),10^{-4}\cdot I\right), \end{array}$$

where $\tilde{g}$ represents the local linear approximation of *g* in Equation (28a).

The observation model simply captures the observation noise

(31) $$\begin{array}{r} p\left(x_{k}|s_{k}\right)=N\left(x_{k}|s_{k},10^{-4}\cdot I\right). \end{array}$$

The internal model is completed with a tight state prior and vague control priors:

(32a) $$\begin{array}{rc} p\left(s_{0}\right) & =N\left(s_{0}|\left(-0.5,0\right),10^{-12}\cdot I\right) \end{array}$$

(32b) $$\begin{array}{rc} p\left(u_{k}\right) & =N\left(u_{k}|0,10^{12}\right). \end{array}$$

**Figure 16:**
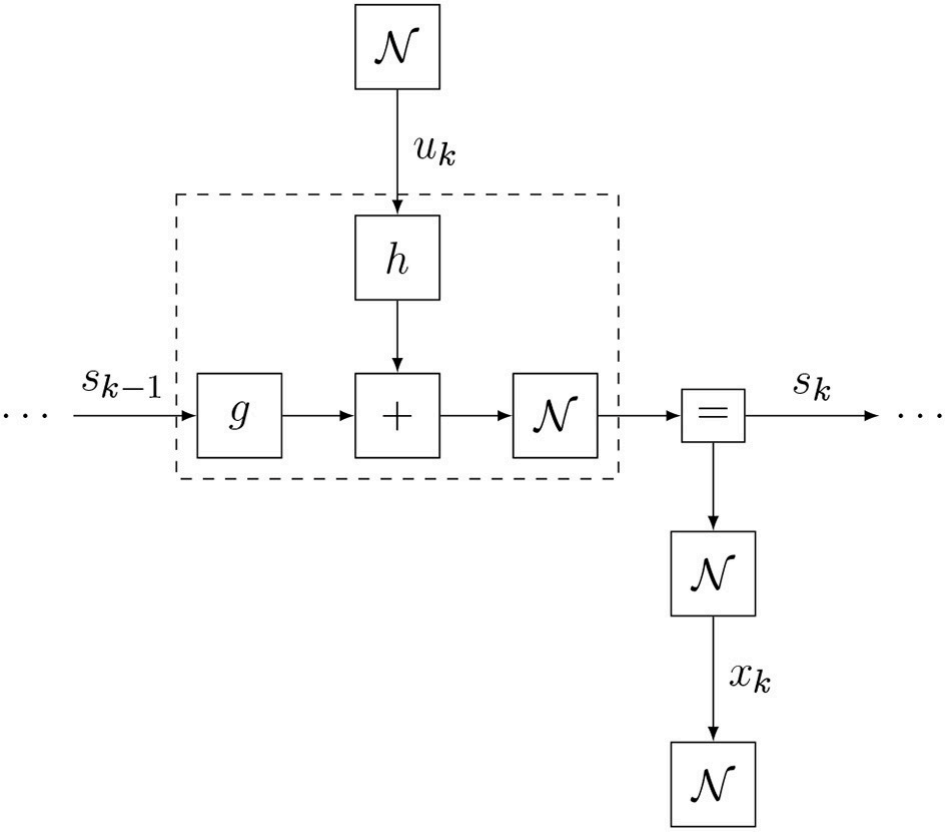
A factor graph visualization of one time slice of the agent's internal model for the mountain car problem (excluding the initial state prior).

These priors specify an accurate belief over the initial state and few prior constraints on control signals. Figure 16 shows one time slice of the agent's internal model.

#### 5.2.3. Simulation Results

**Figure 17:**
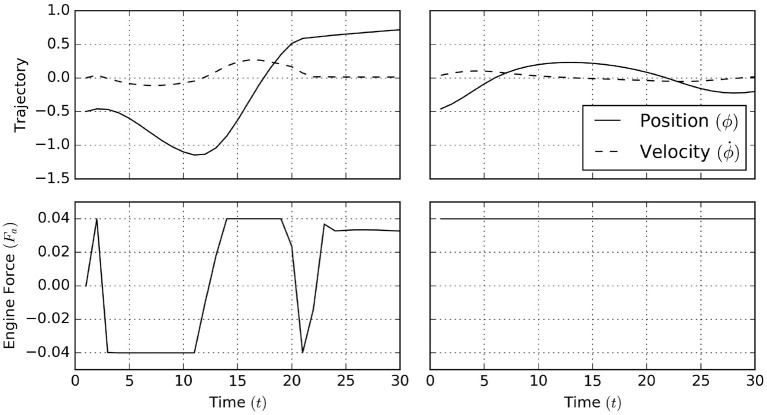
A naive policy in the mountain car task applies a full thrust to the right (right column). This naive policy is unable to make the agent traverse the barrier raised by the gravitational force. In contrast, active inference (left column) automatically infers that the agent should first move left (away from its goal) and subsequently use its momentum (and full forward thrust) to traverse the barrier.

The simulation results^1^ for 30 time steps and with horizon *T* = 20 are shown in Figure 17. Here, a naive policy (right column) with full throttle to the right does not overcome the steepest part of the incline, while the active inference process (left column) infers the need to move left before engaging full throttle to the right in order to reach the desired goal state.

## 6. Related Work

Languages and toolboxes for automated probabilistic inference are increasingly studied in the research literature under the label Probabilistic Programming (PP). Recent state-of-the-art PP toolboxes such as Stan (Carpenter et al., 2017), Edward (Tran et al., 2016), and Infer.NET (Minka et al., 2018) support a broad spectrum of models and algorithms. However, dynamic models incorporate specific structures that may be exploited for improved algorithm efficiency. The SPM toolbox (Friston, 2014) includes specialized routines for simulating active inference processes, but offers limited modeling flexibility. ForneyLab marries flexible model design with automated derivation of efficient structured inference algorithms on dynamic models (Cox et al., 2019). Furthermore, because ForneyLab produces inference algorithms as stand-alone (Julia) programs, these algorithms can be manually optimized before execution.

Agent-environment interactions can be viewed from the perspective of coupled dynamical systems (Beer, 1995). It was recognized early on that a successful regulating agent must include a model of its environment (Conant and Ashby, 1970). Later efforts in the field of model-predictive control used this idea to build controllers for industrial applications (Camacho and Alba, 2013). Various reinforcement learning techniques have also been applied to the mountain car parking problem (Kuss and Rasmussen, 2004; Fürnkranz et al., 2012; Sutton and Barto, 2018), which can be extended to hierarchical contexts as well (Barto and Mahadevan, 2003). A comparison between active inference, risk-sensitive control (van den Broek et al., 2010) and expected utility maximization is provided by Friston et al. (2015). Further comparisons between active inference and reinforcement learning can be found in Friston (2012), Friston et al. (2012), and Friston and Ao (2012).

While the present paper considers a discrete-time formulation of active inference, simulations of active inference (Friston et al., 2009; Pio-Lopez et al., 2016; Buckley et al., 2017) have also been formulated and performed in the continuous-time domain (Friston et al., 2008). In the continuous-time treatment, preferred states are encoded as attracting sets in the dynamical system, and active inference leads the system to these attracting sets over time. While the continuous-time formulation allows for a more standard mathematical treatment, the discrete-time formulation supports explicit reasoning about targets at specific time-points and thereby more easily supports specification of priors for value-seeking behavior (Friston et al., 2015; Parr and Friston, 2017).

## 7. Discussion and Conclusion

This paper has described a message passing approach to automating simulations of online active inference processes, together with an experimental protocol that governs the interactions between the agent and its environment. We have tested our protocol on two synthetic applications, namely the Bayesian thermostat and the mountain car parking tasks. Through these examples we have addressed the questions formulated in section 1, and illustrated how:

1. the proposed experimental protocol defines how to simulate the interactions between an active inference agent and its environment (section 4);
2. The ForneyLab toolbox allows for automatic scheduling of message passing algorithms for variational free energy minimization (section 5) in active inference agents.

The FFG formalism offers a modular decomposition of the internal model definition, allowing flexible model adaptations and intuitive visualization of complex models. Moreover, message passing algorithms for free energy minimization can be automatically derived on the FFG formulation of the agent's internal model. Automated derivation with ForneyLab returns the inference algorithm as a Julia program, which can be customized and executed in context of an experimental protocol.

The proposed experimental protocol formulates the active inference process at each time step as an interplay between updating an internal (generative) model with actions and outcomes (“act-execute-observe”), followed by updating the recognition model (“infer”) with the (statistical) consequences of the change in the generative model. Crucially, the agent and its environment solely interact through the exchange of actions and outcomes.

A current limitation of active inference with ForneyLab is that high-dimensional models may lead to numerical instabilities. Message passing with improved numerical stability is described by Loeliger et al. (2016). Furthermore, the specific message update order as prescribed by the schedule may have an effect on algorithm convergence. However, little theory still exists on optimal scheduling strategies. An interesting idea was mentioned in de Vries and Friston (2017), where it was suggested to approach the scheduling problem as an inference process that is itself subject to the free energy principle.

The presented approach to active inference relies fully on automatable inference methods. This aproach scales in principle to more complex applications that may be of interest to industry as well. For example, the state space models in the examples can be readily extended to hierarchical generative models (Kiebel et al., 2009; Senoz and de Vries, 2018), which have been shown to be quite powerful in modeling real-world dynamics (e.g., Turner and Sahani, 2008; Mathys et al., 2014).

In order to construct hierarchical models, the policy priors may optionally depend on higher-order states, e.g., $p\left(u_{k}|s_{k}^{\left(1\right)}\right)$, which renders prior constraints on control context-dependent. Similarly, target priors may also be made context-dependent, e.g., $p\left(x_{k}|s_{k}^{\left(1\right)}\right)$. Contextual processes may thus influence the agent's behavior by modifying prior statistics, which allows the model design engineer to propose hierarchical and context-aware models. For example, when higher-order states evolve over longer timespans, hierarchical nesting leads to deep temporal models (de Vries and Friston, 2017;Friston K. et al., 2017).

Higher-order dynamics could also be learned by free energy minimization (Ramstead et al., 2018). For example, the current simulations internalize a fixed model of the environmental dynamics. By including a prior belief over the dynamics in the internal model, the agent might learn the environmental dynamics from data (Ueltzhöffer, 2018). This adaptive agent then exhibits epistemic behavior and will take action in order to decrease uncertainty about the environmental dynamics (Friston et al., 2016; Cullen et al., 2018). Moreover, the FFG paradigm supports messages that are computed by local gradient or sampling-based methods (Dauwels, 2007), and even allows for learning complex updates from data with the use of amortization techniques (Stuhlmüller et al., 2013; Gershman and Goodman, 2014). With these techniques, an adaptive agent might learn a rich and accurate model of its environment, leading to more effective behavior.

In summary, the current paper has proposed a scalable approach to automatic derivation of active inference algorithms and a practical view on the implementation of simulated active inference systems. We believe that synthetic active inference holds great promise for future engineering applications.

## Author Contributions

TvdL performed the simulations. TvdL and BdV wrote the manuscript.

### Conflict of Interest Statement

The authors declare that the research was conducted in the absence of any commercial or financial relationships that could be construed as a potential conflict of interest.

## Footnotes

^1^Code for the complete simulation is available at http://biaslab.github.io/materials/ai_simulations.zip.
